# Infective endocarditis following COVID-19 pneumonia: about two cases

**DOI:** 10.11604/pamj.2021.40.152.32071

**Published:** 2021-11-12

**Authors:** Amal El Ouarradi, Aziza Kantri, Khalid Agrad, Ilham Bensahi, Fatimazzahra Merzouk, Zineb Guennoun, Said Makani, Yousra Jebbari, Chafik Elkettani, Mohamed Sabry

**Affiliations:** 1 Department of Cardiology, Mohammed VI International University Hospital, Mohammed VI University of Health Sciences (UM6SS), Casablanca, Morocco,; 2 Department of Anesthesia, Mohammed VI International University Hospital, Mohammed VI University of Health Sciences (UM6SS), Casablanca, Morocco,; 3 Department of Heart Surgery, Mohammed VI University of Health Sciences, Cheikh Khalifa Hospital, Casablanca, Morocco

**Keywords:** Infective endocarditis, COVID-19, heart failure, SARS-CoV-2, case report

## Abstract

Coronavirus disease 2019 (COVID-19) has emerged as a pandemic and public health crisis across the world. The severity of this situation is escalating in certain populations, particularly when the COVID-19 diagnosis may delay the recognition of more dramatic illnesses such as infective endocarditis, which is a dreaded complication in patients with cardiac disease. We report the case of two patients who presented with infective endocarditis initially mistaken for COVID-19 pneumonia, which was responsible for a delay in diagnosis. We discuss the diagnostic difficulties as well as the management of this complication in the COVID-19 era. As a physician, one must remain alert to this dreaded complication, especially in patients with a cardiac history, in order to prevent it, detect it early, and manage it in time.

## Introduction

On March 11^th^, 2020, the World Health Organization declared a global pandemic caused by the 2019 coronavirus (COVID-19). Since then, this disease has affected more than 12 million people in more than 200 countries or regions of the world [1]. Manifestations of COVID-19 infection are dominated by fever, cough, dyspnea, myalgia, and fatigue. Complications include acute respiratory distress syndrome and acute cardiac injury [2]. Bacterial co-infections and superinfections in patients with COVID-19 have been reported [3] and aggravate the disease. We report the case of two patients with cardiac comorbidities who developed infective endocarditis following COVID-19 pneumonia.

## Patient and observation

### Patient 1

**Patient information:** a 26-year-old patient with a history of surgical closure of an interventricular septal defect at the age of 12.

**Clinical findings:** on admission, the patient was tired, with New York Heart Association (NYHA) stage IV dyspnea, orthopnea, febrile with a temperature of 38.9°C, heart rate of 110 cpm, respiratory rate of 40 cpm, blood pressure of 110/60 mmHg, and saturation of 96%, with signs of congestive heart failure: turgidity of the jugular veins, hepato-jugular reflux, and edema of the lower extremities, with diffuse purpuric lesions. Cardiac auscultation revealed a regular rapid rhythm with a gallop and an intense mesocardial holosystolic murmur and an aortic diastolic murmur. Pulmonary auscultation revealed diffuse crackles.

**Timeline of current episode:** the patient reported cough and fever two months before his admission, with a positive polymerase chain reaction (PCR) testing for COVID-19, and negative blood cultures, without signs of endocarditis on echocardiography. The patient was treated at home with azythromycin, amoxicillin, corticoids, vitamins and antiplatelet agents. At first, there was an improvement with resolution of fever and the respiratory symptomatology. But after a few days, the fever resurged associated with a dyspnea stage II to III of the NYHA and edemas of the lower limbs. Therefore, the patient was admitted to the emergency room.

**Figure 1:**
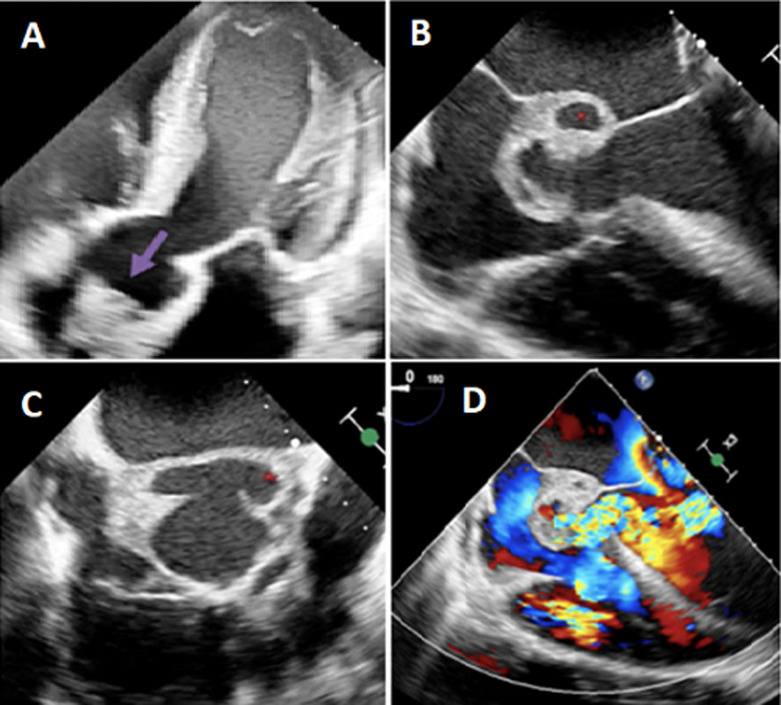
clinical case 1; A) transthoracic echocardiography five chamber view showing a vegetation in the aortic valve (arrow); (B,C,D) transesophageal echocardiography showing abscess in aortic root (star) with Doppler signal showing aorta regurgitation

**Diagnostic assessment:** the electrocardiogram (ECG) showed sinus tachycardia with signs of left ventricular hypertrophy and secondary repolarization abnormalities. The pulmonary computed tomography (CT) scan showed cardiomegaly with bilateral central and peripheral ground-glass lesions (Figure 1). Echocardiography showed a vegetation in the aortic valve, with significant aortic regurgitation and a detergent abscess at the aortic root (Figure 1), with a small residual ventricular septal defect. On the biological workup (Table 1), he had an inflammatory syndrome, the PCR test for COVID-19 was negative and the COVID-19 serology was positive (IGg). Three blood cultures were done, and turned out to be negative.

**Table 1:** relevant laboratory before and after admission and subsequent days

	Normal range	Case 1		Case 2				
		Two months before admission	Day 1	One month before admission	Day 1	Day 3	Day 6	Day 9
Hemoglobin (g/dl)	13-18	11	8.4	11.4	9.2	10	9,6	9
Platelets (103/mm3)	150-400	268	249	250	392	410	402	226
WBC (103/mm3)	4-11	10	19.5	9.5	11.5	43	31	20.2
Neutrophils (103/mm3)	1.4-7.7	7.5	17.6	5.5	9.5	40	29	18.5
TP %	70-100	88	80	35	45	60		
CRP hs (mg/l)	<5	10	135	4	35	113	40	9.8
Procalcitonin (ng/ml)	<0.5	0.02	0.56	0.03	0.15	60	28	8.6
Ferritin (ng/ml)	20-200	30	330	100	349			
LDH (UI/l)	80-230	180	280	190	295			
D-dimer (ngFEU/ml)	<500	400	8262	350	742			
Haptoglobin (g/l)	0.32-1.9		4.58					
Troponin T us (ng/mL)	<0.14	0.04	0.11	0.01	0.005	0.10		
NT-Pro-BNP (pg/ml)	<300	250	5400	300	3389			
AST (U/L)	<50	30	245	15	13	47		
ALT (U/L)	<50	34	236	15	15	30		
Urea (g/l)	0.17-0.49	0.3	0.46	0.13	0.13	0.23		
Creatinin (mg/l)	6-11.7	6.7	7	6.5	6.5	13.9		
CBU test			Leucocytury without bacteriury					
Nasopharyngeal swab		Positive	Negative	Positive	Negative			
COVID-19 serology			Positive (IGg, no IGm)		Positive (IGg, no IGm)			
Blood culture		Negative	(3) Negative	Negative	Positive; enterococcus; streptococcus equinus			

ALT: alanine aminotransferase; AST: aspartate aminotransferase; CRP hs: C- reactive protein hs; LDH: lactate dehydrogenase; NT- pro BNP: N-terminal pro brain natriuretic peptide; WBC: white blood cell count

**Diagnosis:** aortic infective endocarditis and aortic abscess with massive aortic insufficiency.

**Therapeutic interventions:** symptomatic treatment of heart failure, associated with antibiotic therapy based on vancomycin and gentamicin, an urgent surgery has been proposed (resection of the abscess with aortic valve replacement).

**Follow-up and outcome of interventions:** however, the patient presented quietly a state of shock with multivessel failure and died.

**Patient perspective:** before his death: “I have the hope to be cured”.

**Informed consent:** the family of the deceased has given informed consent.

### Patient 2

**Patient information:** a 50-year-old female patient, followed for atrial fibrillation and rheumatic valvulopathy under anti vitamin K.

**Clinical findings:** the patient presented with chills, profound fatigue, stage II dyspnea, palpitations and severe headache.

**Timeline of current episode:** the patient reported one month before her admission a moderate form of COVID-19 pneumonia with dry cough and positive PCR test for COVID-19, with negative blood cultures without signs of endocarditis on echocardiography. She was treated at home with azithromycin, amoxicillin, vitamin, and corticoids. The evolution was marked by the resolution of the cough with clinical aggravation, an alteration of the general state and the appearance of important headaches.

**Figure 2:**
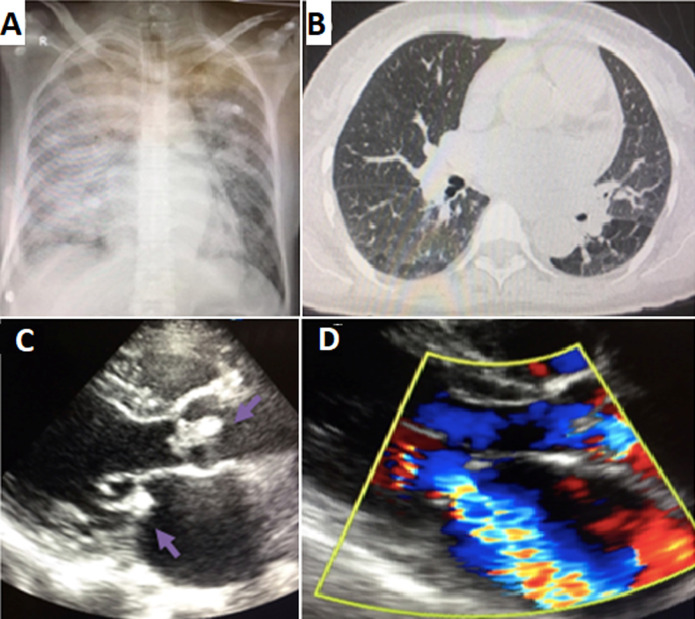
clinical case 2; A) chest X-ray showed a bilateral diffuse alveolar syndrome and cardiomegaly; B) thoracic CT scan a cardiomegaly with the presence of peripheral, central and bilateral ground glass lesion; C) transthoracic echocardiogram: parasternal long axe view showing a vegetation in the aortic and mitral valve (arrows); D) Doppler signal showing mitral regurgitation

**Diagnostic assessment:** the clinical examination on admission found an apyretic patient with a temperature of 36.6°C, a heart rate of 130 cpm, a respiratory rate of 30 cpm, a blood pressure of 110/60mmHg, and a saturation of 98%, no signs of right heart failure. The cardiac auscultation found an irregular rhythm with a murmur of mitral insufficiency. The pulmonary auscultation found diffuse crackles. The neurological examination was normal. On ECG, there was atrial fibrillation with electrical signs of left ventricular hypertrophy. Her chest X-ray showed a bilateral diffuse alveolar syndrome and cardiomegaly. The thoracic CT scan showed a cardiomegaly with peripheral, central and bilateral ground glass lesions. On echocardiography, there were mitroaortic vegetations with aortic and mitral regurgitation (Figure 2). Because of the strong headache, a cerebral magnetic resonance imaging (MRI) was done and showed a temporoparietal hemorrhagic stroke. Her biological workup (Table 1) showed a moderate inflammatory syndrome at the beginning that rapidly increased after the third day. The PCR test for COVID-19 was negative. The blood cultures were positive isolating an Enterococcus and a Streptococcus Equinus.

**Diagnostic:** we concluded that the patient had a mitroaortic infective endocarditis complicated by heart failure and hemorrhagic stroke.

**Therapeutic interventions:** anticoagulation stopped, with symptomatic treatment of heart failure, associated with antibiotic therapy based on vancomycin and gentamicin, cardiac surgery (mitroaortic valve replacement) proposed one month after neurological stabilization.

**Follow-up and outcome of interventions:** neurological aggravation and death.

**Patient perspective:** before his death: “I have the hope to be cured”.

**Informed consent:** the family of the deceased has given informed consent.

## Discussion

SARS-CoV-2, the cause of the COVID-19 pandemic has significantly impacted cardiovascular healthcare. Patients with pre-existing cardiovascular disease are at higher risk of morbidity and mortality. The virus may affect the heart directly and indirectly with clinical syndromes of acute myocardial injury, myocarditis, acute coronary syndromes, heart failure, arrhythmias, and venous thromboembolism. Some therapeutics under investigation for COVID-19 may also have adverse cardiac effects [4].

Infective endocarditis, known since the year 1900, is a difficult and complex disease, it is defined by a colonization of the endocardium by microorganisms most often bacterial [5]. It is frequently contracted in the context of health care, and more than half of the cases occur in patients with known heart disease. Despite optimal care, mortality approaches 30%. The challenges posed by infective endocarditis are significant. It is heterogeneous in terms of etiology, clinical manifestations and evolution [6]. It may present with general signs such as fever, asthenia, or with signs of heart failure or with systemic embolic or infectious complications. In the biological work-up, the inflammatory syndrome is almost constant, with an increase in N-terminal natriuretic peptide of the pro-B type, and in D dimer, which can simulate a COVID-19 infection [7]. The concomitant association of infective endocarditis and COVID-19 remains rare, at present, a few rare cases have been reported in the literature. The most frequently described endocarditis in severely ill COVID-19 patients is the right sided endocarditis in the tricuspid valve. It is particularly found in patients with prolonged intubation where multidrug-resistant Gram-negative bacteria are frequently observed probably reflecting a nosocomial infection [8-10]. Rare cases of infective endocarditis following COVID-19 infection have been reported, describing the difficulties of managing this condition [9]. In our two patients at high risk of infective endocarditis because of their cardiac history, the occurrence of an infective endocarditis in the following of a COVID-19 pneumopathy can have as hypothesis a bacterial pulmonary superinfection in the following of COVID-19 as a portal of entry responsible for an infective endocarditis, with the taking of corticosteroids weakening the immunity and predisposing to serious infections, but a direct causal link is not sure. Also, COVID-19 masked the cardiac symptomatology, especially with antibiotic use, delaying the early diagnosis to the point of very advanced infection.

According to Van Camp *et al*. and Escolà-Vergé *et al*. the incidence of infective endocarditis has decreased during this period of COVID-19 and this is due according to their report to the change of health system and to the containment measures imposed by the authorities in this pandemic of COVID-19, responsible for a delay in the diagnosis of this deadly pathology [11,12], but Havers-Borgersen *et al*. report in a Danish cohort that no significant difference in the incidence of infective endocarditis admissions during the national lockdown due to the COVID-19 pandemic was found [13].

The diagnosis of infective endocarditis is difficult, it is facilitated by the use of the standardized Duke classification, which combines two major criteria (microbiology and imaging) and five minor criteria (fever, predisposing cardiopathy, vascular phenomena, immunological phenomena, microbiological evidence) [14]. Concerning the treatment, the decision to start antibiotherapy must be individualized and discussed with the endocarditis team while waiting blood cultures results. In hemodynamically unstable patients with clinical presentation in favor of infective endocarditis, empirical antibiotic therapy can be initiated (grade IIb) [6]. For patients who are clinically stable, antimicrobial therapy may be delayed while awaiting blood culture results and can be adjusted according to the antibiotic sensitivity spectrum results [14]. In a patient with heart failure or mechanical complication with severe aortic or mitral insufficiency and large vegetations, related to EI, surgery is the best option, unless there is a neurological complication, when cardiac surgery must be balanced with perioperative risk and postoperative prognosis [14].

## Conclusion

Infective endocarditis remains a deadly disease and early diagnosis is the corner stone for early treatment and avoiding unnecessary complications. In this current context of COVID-19, the medical core must remain attentive to this dreaded complication especially in patients with a cardiac history in order to prevent it, detect it early, and manage it.
